# Defining the concept of family caregiver burden in patients with schizophrenia: a systematic review protocol

**DOI:** 10.1186/s13643-019-1182-6

**Published:** 2019-11-26

**Authors:** Zahra Tamizi, Masoud Fallahi-Khoshknab, Asghar Dalvandi, Farahnaz Mohammadi-Shahboulaghi, Eesa Mohammadi, Enayatollah Bakhshi

**Affiliations:** 1 PhD Candidate in Nursing, University of Social Welfare and Rehabilitation Sciences, Tehran, Iran; 2 Professor, Head of the Department of Nursing, University of Social Welfare and Rehabilitation Sciences, Tehran, Iran; 3 Associate Professor Emeritus, Department of Nursing, University of Social Welfare and Rehabilitation Sciences, Department of Nursing and Midwifery, Tehran Medical Sciences, Islamic Azad University, Tehran, Iran; 4 Professor of Iranian Research Center on Aging, Nursing Department, University of Social Welfare and Rehabilitation Sciences, Tehran, Iran; 5 Professor, Department of Nursing, Faculty of Medical Sciences, , Tarbiat Modares University, Tehran, Iran; 6 Associate Professor, Department of Biostatistics, University of Social Welfare and Rehabilitation Sciences, Tehran, Iran

**Keywords:** Family caregiver, Burden, Schizophrenia, Protocol

## Abstract

**Background:**

Since the deinstitutionalization policy, in psychiatric hospitals, the care of patients with schizophrenia was left to their families which has been imposing a heavy burden on them. Family caregiver burden could have consequences for caregivers, patients, and the society. There is very little consensus on the definition and dimensions of the caregiver burden, which leads to a lack of consistency in the results of research. Thus, the present study was aimed to redefine the family caregiver burden of patients with schizophrenia.

**Methods:**

The databases PubMed, Scopus, Web of Science, MEDLINE (Via Ovid), ProQuest, SCI, Magiran, SID, and IranDoc will be searched from 1940 to 2018 using subject headings and appropriate terms in both Farsi and English languages. Also, gray literature and the reference list of included articles will be used to offer an appropriate definition of the family caregiver burden in patients with schizophrenia. Two independent reviewers will participate in study selection, data collection, and quality assessment steps. The result will be presented in tabular form, and meta-synthesis will be performed.

**Discussion:**

The result of this systematic review will help present the comprehensive definition of the family caregiver burden in patients with schizophrenia according to its evolutionary trend.

**Systematic review registration:**

PROSPERO CRD42018099372

## Background

The caregiver burden has common characteristics in physical and mental diseases, and caregivers often experience symptoms such as anxiety disorders and depression, as well as many economical and occupational problems. However, different pathologies make specific effects on caregivers through the symptoms and social reaction to them, so these differences create special needs for care of patients [1].

Based on the studies carried out by the World Health Organization, schizophrenia is one of ten diseases leading to the loss of ability in individuals [2]. Schizophrenia is a significantly disabling and chronic psychiatric disorder that affects all major domains of a patient’s life. The prevalence of this disorder is approximately 3–6.6 of 1000 persons [3]. The World Health Organization estimated that 29 million persons were affected by schizophrenia worldwide [4]. In an epidemiological study of psychiatric disorders in Iran, the prevalence of psychotic disorders has been reported to be 0.89%, with the prevalence of schizophrenia to be 0.6% [5].

The majority of mental disorders often imposes a heavy burden on caregivers; however, among these disorders, schizophrenia attracts more attention not only due to the deterioration of the patient’s individual and social performance and the symptoms that affect the caregivers’ quality of life but also because of the nature and the early breakout of the disorder [6].

The caregivers of patients with schizophrenia experience a heavier burden than other psychiatric disorders [4, 7, 8]. The family caregiver burden impresses the physical and mental health [2, 9], social relationships [7, 9–11], and the financial life [7, 9, 10, 12] of caregivers. Also, it is associated with psychological morbidity [7], less leisure time, workload, and burnout of caregivers [13]. They also experience a feeling of frustration, anger, embarrassment, fear, sadness, and stress because of the behavior of patients [14, 15], as well as a negative attitude toward the patient [10].

Several review studies have been conducted on the family caregiver burden in patients with schizophrenia. In this regard, Glanville and Dixon (2005) investigated the family caregiver burden as well as family treatment approaches and services used in the families of patients with schizophrenia. They stated the complex nature and multidimensionality of the family caregiver burden were neglected; also, family treatment approaches focus more on patient well-being and pay less attention to caregivers’ appraisals [16]. Schulze and Rossler (2005) reviewed the development of burden scales and family interventions. They reported there is no consensus on dimensions of instruments to measure the caregiver burden and also there are some deficits of burden scales which restrict their usage in clinical practices [17].

Awad and Voruganti (2008) examined the historical development of this concept, its definitions, and other related factors [18]. Also, the results of the study by Macleod et al. (2010) indicated a combination of education, mutual support, and coping strategies delivered within an intensive community program can reduce the caregiver burden and also improve their mental health [19]. Chan (2011) reported that despite cultural diversity, the family caregivers’ burden is a global issue and it is experienced by family caregivers of patients with schizophrenia in various parts of the world, so the designing and implementation of family-centered programs are essential needs for family caregivers [4]. Also, Seeman (2013) showed women whose husbands are suffering from schizophrenia bear a lot of burden. The caregiving burden causes much marital discord; in this regard, marital support and counseling are needed more than ever [20].

Caqueo-Urízar et al. (2014) studied the family caregiver burden and related factors in patients with schizophrenia. Also, they reported the use of different theories for description for the family caregiver burden is a reflection of the complex nature of this concept [21].

Miller et al. (2014) reported the human burden of schizophrenia is beyond the patients and caregivers. Caregivers of patients with schizophrenia suffer from different kinds of physical, psychological, emotional, social, and financial problems in their lives [13]. Chong et al. (2016) investigated the economic burden of schizophrenia and explained schizophrenia is associated with both direct and indirect financial burdens [22]. In addition, the results of Shiraishi and Reilly’s study (2017) showed family members of schizophrenia patients experience traumatic events at the onset of the disease. Then, they experience negative impacts such as uncertainty, unpredictability behaviors, stigma, limitation of personal and social resources, family disruptions, and conflicts in interpersonal relationships during the continuous caregiving. Furthermore, they experience positive aspects of caregiving such as compassion, self-confidence, and personal growth in the same caregiving cycle [23].

To better understand and describe the consequences of the care of patient with schizophrenia, the concept of the family caregiver burden was used. Several definitions of this concept are presented in these studies; however, there is no agreement on its dimensions and attributions. In addition, the classification of the burden into objective and subjective components was criticized because this division leads to neglect the burden multidimensional and complex nature. Furthermore, the antecedents and the consequences of the caregiver burden are not thoroughly described.

The family caregiver burden is a multidimensional concept with dimensions comprising social, emotional, and financial issues as well as relationships with a care receiver and the shortage of time; however, there is little agreement on the major dimension or the way they are interrelated [24, 25]. Schene et al. (1996) believe the reason for the researchers’ little agreement on the various dimensions of the family caregiver burden is related to the definition of the burden and the method of measuring the subjective and objective burdens so that these differences in the opinion have operationally affected on the measurement of the special dimensions of the family caregiver burden [25]. In this regard, Annisa et al. (2016) argue the conceptualization and clear definition of the burden are very difficult and what has so far been defined as the burden is in fact the stressors [26]. Moreover, in many studies, some words like stress, distress, and burnout have interchangeably been used for the word “burden” that are not distinguishable from one another [27, 28] Therefore, the use of interchangeable words for the concept of burden has seriously hidden the real meaning of the burden [28].

Considering there is no clear definition of the concept “the family caregiver burden” [25] and lack of a consistent conceptualization and operational definition for it in research, the need to clarify the concept of burden and to understand its relevant attributes would help explicate its usefulness in practice and research [27], so the present study aimed at clarification and comprehensive description of the family caregiver burden in patients with schizophrenia will be conducted.

## Methods/design

This protocol has been written in accordance with the recommendation of Preferred Reporting Items for Systematic Review and Meta-Analysis (PRISMA-P) 2015 statement [29].

### Review question

What is the definition of the family caregiver burden in patients with schizophrenia? What are its dimensions? What are its underlying attributions, antecedents, and consequences?

### Systematic review objectives

The review will meet the following research objectives:

#### Primary objectives

To summarize the definition of the family caregiver burden in patients with schizophrenia.

#### Secondary objectives

1. Determining the dimensions of the family caregiver burden in patients with schizophrenia.
2. Determining the attributions of the family caregiver burden in patients with schizophrenia.
3. Determining the antecedents and consequences of the family caregiver burden in patients with schizophrenia.

### Registration of protocol

The systematic review protocol has been registered in PROSPERO with registration number CRD42018099372.

### Inclusion and exclusion criteria

#### Study characteristics

In this systematic review, varieties of studies, representing at least one definition of the family caregiver burden in patients with schizophrenia, comprise observational studies (case study, case series study, cross-sectional study, case-control study, and cohort study), interventional studies (true experimental and quasi experimental studies, randomized and non-randomized studies, field trials, and community interventional studies), and qualitative studies, and also review studies will be included.

#### Types of participants

In this systematic review, the studies whose research subjects have been the caregivers of patients with schizophrenia, have experienced at least 1 year in caring for a patient, and are at least 18 years old or belong to one of the age groups or at least male or female in gender will be included.

#### Setting and time frame

The period of conducting the studies is from 1940 to 2018, without any restriction on the place of study.

#### Report characteristics

With respect, articles in English or Farsi languages and articles published or are in press will be included in the study.

#### Information resources

The search resources include electronic databases, various types of gray literature, a reference list, the registration system for trial studies, and manual search. The electronic resources included are PubMed, Scopus, Web of Science, MEDLINE (Via Ovid), ProQuest, SCI, Magiran, SID, and IranDoc. The triple-phase search method was used to identify relevant terms. So, some of the most relevant articles were selected in order to find closely connected terms or phrases. In the second phase, related databases or other unofficial sources were searched using the keywords obtained in the first phase. In the last step, the reference list of related articles selected in the previous step was checked to select suitable terms. The search strategies will be developed in PubMed, and then, the same syntax will be applied to other databases. In addition, other sources such as ProQuest, IranDoc, Google Scholar, and Web of Science will be searched for gray literature such as dissertations, theses, and posters.

#### Search strategy

The initial search method will be based on the syntax produced in the database of PubMed as follows: (((caregiv*) [tiab] OR career [tiab]) AND burden [tiab] AND Schizophrenia [tiab] OR "Schizophrenia Spectrum disorder"[tiab] AND 940/01/01[PDAT]: 2018/06/01[PDAT]). In addition, equivalent keywords will be used for searching Persian databases (Additional file 1).

### Data management

The related data will be extracted by two researchers independently and will be recorded in data sheets subsequently. A third party will review the two data sheets. The possible disagreements between the two researchers will be discussed with the whole team. If no solution is obtained, the researchers will contact the authors of the paper to make the final decision.

### Selection process

The search process will be completed using the appropriate syntax developed in PubMed, then, duplicate articles will be omitted. Two members of the research team will independently review the titles and abstracts of the articles to evaluate their eligibility. The references will be categorized into three groups: “relevant,” “irrelevant,” and “uncertain” subcategories. Then, two researchers will independently review the full text of all the references under the “relevant” and the “uncertain” categories based on inclusion criteria. Any disagreement will be noticed and resolved through discussion among these two researchers so as to achieve consensus. If they could not reach any consensus, a third researcher of the research team will arbitrate. Also, if multiple reports of a study will be identified, they will be considered as one study but reference will be made to all the publications. If any discrepancies are found between these multiple reports, they will be marked to contact the authors for clarification.

### Data extraction

The data will independently be extracted from the articles by two researchers and will be imported into a data extraction form. In this section, the entire research group will assess cases of disagreement between them and the final decision will be made. 

In each article, data such as the title, years of publication, place of research, type of discipline, research methodology, target population, samples’ demographic information (age, gender, etc.), sample size, significant related variable to burden, definitions of the family caregiver burden, dimensions of family caregiver burden, and instruments in data collection will be collected (Table 1).

**Table 1:** Data extraction form

Code of article	Title of article
	Years of publication
	Place of research
	Type of discipline
	Research methodology
	Target population
	Age or age group of sample
	Sex of samples
	Sample size
	Significant related variable to burden
	Definition of family caregiver burden concept
	Dimension of family caregiver burden concept
	Scale of family caregiver burden in data collection

### Risk of bias assessment

The studies will be critically appraised for appropriateness of study design to the research objective, quality of the intervention, data collection, analysis method, interpretation, quality of reporting, generalizability, kind of bias, confounders, and attrition. Then, studies will be categorized to the findings uncertain, high, or low [30].

Cochrane Collaboration tool (ROB) will be used for the risk of bias assessment in controlled trial studies [31]. Also, the non-controlled trials, the quasi-experiments, and systematic reviews will be assessed by risk of bias in non-randomized studies of interventions (ROBINIS-I) tool [32]. In addition, Qualitative Assessment and Review Instrument will be used for appraising of qualitative studies [33]. This step will be conducted by two members of the research team independently.

### Data synthesis

In this study, data will be analyzed by a narrative approach, specifically thematic synthesis. The analysis will comprise two phases. For the first review question, we will present the domains of the definitions of family caregiver burden in schizophrenia patients in the two subcategories of study design and the publication year. Finally, we will report various definitions of family caregiver burden into a table based on their importance and the degree of satisfaction that will be determined by their quality of study and reliability of results.

For the other review questions, the thematic analysis will be used and it will comprise three phases. First, each article will be considered as a unit of analysis and will be read several times by a member of the research team to understand the general meaning of behind the data. The words, sentences, or phrase related to family caregiver burden will be identified as a meaning unit considering study objectives. The primary codes will be developed from the meaning unit and will be check by a second researcher. The research team will make the final decision if there will be disagreement in coding. In the second stage of the analysis, the primary codes will be categorized into more definite categories or subcategories based on similarities and differences. Finally, themes with regard to the underlying meanings in the studies were extracted. The themes are entered into the columns and the codes are entered into the rows of a table. The constant comparison of data with data and data with codes will be used to facilitate comparison within and between studies. At last, the descriptive and analytical themes will be recorded based on the agreement of the entire research team. The final report will be prepared based on the Preferred Reporting Items for Systematic Reviews and Meta-Analyses (PRISMA).

## Discussion

Because of the lack of clarity in the concept of the family caregiver burden and the alternate usage of surrogate terms such as pressure, distress, tension, and burnout instead of burden [28, 34], as well as the lack of a clear operational definition for this concept, leading into unreliability in study results [27], the research cannot be conducted in family systems and other relevant fields [27, 35]. Therefore, the results of the current systematic review can lead to the clarification and redefinition of the family caregiver burden in patients with schizophrenia and its dimensions. In addition, the results of the present study can be used as a basis in designing specific tools for appraisal of the family caregiver burden in patients with schizophrenia.

## Supplementary information

**Additional file 1: Table S1.** The final syntax.
