# Supplementary material for: Defining the concept of family caregiver burden in patients with schizophrenia: a systematic review protocol
Source: Syst Rev. 2019 Nov 26;8:289. doi: 10.1186/s13643-019-1182-6 (PMC6878682; doi:10.1186/s13643-019-1182-6)
Supplement: Supplementary file 1 — Additional file 1: Table S1. The final syntax. [file 13643_2019_1182_MOESM1_ESM.docx]

| **Table S1: Search Strategies** | |
| --- | --- |
| **Database** | **syntax** |
| PubMed | [(((caregiv*) [tiab] OR career [tiab]) AND burden [tiab] AND Schizophrenia [tiab] OR "Schizophrenia Spectrum disorder"[tiab] AND 940/01/01[PDAT]: 2018/06/01[PDAT]](https://www.ncbi.nlm.nih.gov/pubmed?term=(caregiver%20OR%20career%20OR%20(caregiver%20AND%20spouse)%20OR%20%22spouse%20caregiver%22%20OR%20(caregiver%20AND%20family)%20OR%20%22family%20caregiver%22)%20AND%20burden%20AND%20(schizophrenia%20OR%20%22Schizophrenia%20Spectrum%20disorder%22%20OR%20(schizophrenia%20AND%20paranoid)%20OR%20(schizophrenia%20AND%20disorganized)%20OR%20(schizophrenia%20AND%20catatonic)%20OR%20(schizophrenia%20AND%20childhood)%20OR%20(schizophrenia%20AND%20residual)%20OR%20(schizophrenia%20AND%20undifferentiated)%20OR%20%22Paranoid%20Schizophrenia%22%20OR%20%22Disorganized%20Schizophrenia%22%20OR%20%22hebephrenic%20schizophrenia%22%20OR%20%22Catatonic%20Schizophrenia%22%20OR%20%22Childhood%20Schizophrenia%22%20OR%20%22residual%20schizophrenia%22%20OR%20%22undifferentiated%20schizophrenia%22)%20AND%201940/01/01%5bPDAT%5d%20:%202017/07/23%5bPDAT%5d&cmd=correctspelling)) |
| Scopus | TITLE-ABS-KEY ((caregiv* OR career) AND burden AND (schizophrenia OR "Schizophrenia Spectrum disorder")) |
| Web of Science | TS=(( caregiv* OR career) AND burden AND (schizophrenia OR "Schizophrenia Spectrum disorder")) |
| MEDLINE (Via Ovid) | ((caregiv* OR career) and burden and (schizophrenia or 'Schizophrenia Spectrum disorder')).af |
| ProQuest | ALL((caregiv* OR career) AND burden AND (schizophrenia OR ("Schizophrenia Spectrum disorder"))) |
| SCI | (caregiv* OR career) AND burden AND Schizophrenia OR ("Schizophrenia Spectrum disorder") (ALL FIELD) |
| Google Scholar | allintext: ((caregiv* OR career) AND burden AND (schizophrenia OR "Schizophrenia Spectrum disorder")) |
